# Linear Drive Based on Silicon/Ethanol Composite

**DOI:** 10.3390/polym13162668

**Published:** 2021-08-10

**Authors:** Tomasz Kapłon, Andrzej Milecki

**Affiliations:** Institute of Mechanical Technology, Faculty of Mechanical Engineering, Poznan University of Technology, Pl. M. Skłodowskiej-Curie 5, 60-965 Poznan, Poland; andrzej.milecki@put.poznan.pl

**Keywords:** silicon/ethanol actuator, smart materials, phase change material, positioning

## Abstract

The paper presents a concept of an actuator, based on a silicon/ethanol composite placed in the brass bellows. Such actuator is operating based on a change in the physical state of ethanol, which is enclosed in bubbles surrounded by a matrix of silicone rubber. In this paper, the prototype of the actuator is described, and a series of its test results, in the open and closed loops, are presented. Two laser distance-sensors, with different accuracies, were used as a source of the feedback signal. During the investigations the temperature of the actuator was also measured. This has allowed us to determine the delay in heat flow from the heater to the composite. In the closed loop, P- and PI-type controllers were used in the drive positioning experiments. It was discovered that in the closed loop control, it was possible to achieve a positioning error of less than 200 µm. During the tests, the temperature inside the drive and the ambient temperature were also measured. In order to improve the dynamics of the drive, a small fan was used, controlled by the automation system. It allowed us to shorten the time to return the drive to its starting position. The results of frequency tests of the drive have also been presented.

## 1. Introduction

Smart actuators are made of materials that can change their dimensions in response to change of environmental conditions. Such actuators have no mechanical moving parts and can perform movements similar to human muscles at the nano-, micro- and macro-scale. A variety of smart materials are available today, and among them, silicon-based ones appear to be very attractive for practical applications and research.

A change in the physical state of a material is often associated with a significant volume difference. This phenomenon has been used in the construction of a group of smart actuators. The most common example of a thermal phase change actuator filled with a solid–liquid phase change material is a paraffin wax actuator. In this actuator, the paraffin changes its state from solid to liquid. It can generate quite high forces, in the range of thousands of Newtons and, for volume expansion, of about 20%. The article [1] describes the use of paraffin wax (SIGMA-ALDRICH mp 58–62 °C), which shows a free expansion of the volume by about 15% at 75 °C, compared with 25 °C. Such material is characterized by moderate melting temperature in a range of 78 °C, which is easily obtainable. Therefore, paraffin is used in fine mechanical actuators with a shaft to produce linear motion. The paraffin is usually hermetically sealed in a bellows [2] or is located in silastic tube [3].

The literature also describes cylinder piston actuators with paraffin wax; an example is presented in work [4]. The length of its cylindrical housing is 58 mm. The actuator stroke is 25 mm and its maximum force reaches 160 Newtons. Kabei et al. [3] have designed a cylinder piston actuator which has a paraffin chamber 90 mm long and 2 mm diameter. Without the load, the actuator’s maximum stroke is about 10 mm. However, this stroke decreases slightly under a load of 10 N.

In paper [5] a prototype of actuator is described, which is based on two bonded circular diaphragms. The actuator with the width of 68 mm and a thickness of 75 μm obtains a maximum deflection of 90 μm with a load of 3 N.

Unfortunately, the drives which use phase change materials like paraffin wax are characterized by a low speed of movement and thus also low dynamic response [6,7], which is their serious drawback. The problem of the mentioned-above actuators, is also the relatively low operating temperature. If it is exceeded, the drive may be destroyed.

In paper [8], a new principle of actuators working successfully at 180 °C, which utilize liquid-vapor transition of water, is proposed. The use of triethylene glycol as the working fluid is described in [9,10]. This material has a boiling point of 287 °C. The proposed actuator generates 1.67 mm maximum displacement and it can be utilized in high temperature environments, up to 300 °C.

In recent years, the application of composite materials in actuators has been proposed. In these solutions, the active material, which changes its physical state, is surrounded by an elastic matrix [11,12,13,14]. These categories of materials include silicone/ethanol composites. Such type of material consists of bubbles filled with liquid ethanol surrounded by a silicone rubber. During heating the transformation temperature is reached, in which ethanol starts gradually to change from liquid to gas. The resulting pressure inside the bubbles causes the entire material to expand. This transition is accompanied by a large increase in actuator specific volume. In order to generate displacement, the vapor pressure of ethanol must be higher than the pressure inside the bubbles. For this reason, if the pressure inside the bubble increases, the transformation requires heating to maintain higher temperature to continue the deformation. During cooling, ethanol returns to the liquid state, and the entire material returns to its original dimensions, due to the action of elastic forces of the silicone matrix [15,16]. In [15], the authors describe a silicone/ethanol elastomer composite, suggested as an innovative actuators. They have investigated and assessed the functional properties of this novel approach to soft actuation. In the initial tests, silicone/ethanol composites containing 20 vol.%, 10 vol.%, 5 vol.% and 0 vol.% of ethanol were prepared. The same materials was used in the actuator they built. Mechanical tests have been performed in order to determine which composition has the best output force and strain characteristics [15,16]. For higher ethanol content, the authors have found that there are problems with the correct mixing of ethanol with silicone [15]. In a block-force test, the samples of a composite measuring 50 mm in length and 20 mm in diameter were inserted into an aluminum capsule and have worked as a unidirectional actuator. It was found that, for higher amounts of ethanol, the force grows linearly. For a sample with 20% ethanol content, its maximum force of 950 N has been obtained in the linear operating range at 155 °C. It has been found that, for 20 vol% ethanol, the sample deviation from linearity response starts to occur at about 913 + 85 N, for 10 vol% at 680 + 70 N and for 5 vol% at 394 + 93 N [15]. Because linear behavior is preferred for controlled actuation, the composition with 20% of ethanol was confirmed as the most suitable [15]. In the supplementary materials of paper [16] the response of specimens with content of ethanol 0–33% to heat was investigated. It was found that for specimen with 20% and 33%, the actuation expansion response was similar, but for 20% ethanol, specimen results were more repeatable and that this specimen had a longer work life. In paper [15], 55–85 °C has been determined as the proper temperature range for investigations based on differential scanning calorimetry (DSC) and thermogravimetric analysis (TGA). In such temperature the most significant actuation takes place and the material can work without undergoing rapid degradation [15]. In this range, the force of 130 N has been achieved for the tested sample for a block-force test. DSC and TGA investigations have shown also that for a higher amount of ethanol there are greater differences compared to the baseline for silicone alone [15].

In paper [15], an artificial muscle specimen has been also described. It consists of the material-actuator with embedded Ni-Cr resistive wire encased in a net, inserted into a braided mash sleeve. The measured maximal blocking force within the linear range was about 150 N. This value is 1100 times higher than the material actuator’s own weight force. The muscle maximal displacement for the 50 mm long specimen was 13 mm, which means axial contraction of 26%.

The commonly applied methods of increasing temperature of the composite materials used as actuators are mostly based on resistance heating. Usually, the material is heated with a heater in the form of a spiral, made of resistance wire embedded in the material. The possibility of heating through an elastic core made of conductive polymer was tested in [17]. In [18] the use of special fabric for heating was presented. The prototype of actuator that uses induction heating is successfully applied, and its investigations are described in the paper [19].

Compared to metallic materials like thermal shape memory alloys (TSMA), characterized by a maximum deformation of up to 9% [20,21], the composite ethanol/silicon can achieve a much greater deformation [16]. Additionally, the force generated by the composite discussed in mentioned above papers, can reach a value of several tens of Newtons, while typical force values for TSMA actuators are only several N. Additional advantageous features of the composite are low density ranging around 1 g/cm^3^, and the low price of the ingredients from which it is made. Important disadvantages are: low dynamics characterized by low operating time, smaller than SMA alloys, and greater exposure to degradation when over-heated [15]. Depending on the design, the elongation and recovery cycle time can last as much as several hundred seconds. So far, the possibilities of the silicone-ethanol composite applications have been investigated only in terms of the forces and displacements that can be generated. These studies have largely been carried out on objects that were modified McKibben actuators.

Control of soft actuators is a difficult challenge, especially when they are applied or are the basic structure in multi-axis soft robots. Control of such systems should be based on both advanced methods and morphology of soft materials. The interesting example is the development of adaptive 4D-printed systems [22], which, in order to obtain the assumed parameters, has to be equipped with a set of different elements for measurement of various physical and mechanical properties such as stress, strain, deformation and acceleration. These elements are used as a source of feedback information to the controller unit. The active controller regulates the power in order to manipulate the material’s heating behavior. In the control the changes in resistance caused by the changes in strain or temperature are taken into account. The controller has used a model-based techniques in conjunction with self-learning methods.

The paper [23] presents the review of modelling, fabrication and control of 3D/4D-printed soft pneumatic actuators (SPA). The bending curvature and blocking force of the 3D/4D printed SPAs is predicted using the analytical kinematic modelling. The FEA method is used for dynamic modelling of hyper-elastic materials and various energy functions. Also, the sensors and the control methods which could be applied are analyzed. Finally, the future directions and challenges of SPA actuators are proposed. The control methods used for soft robots can be grouped into two main categories: static and dynamic [24,25]. In dynamic controllers the configuration space or task space variable velocities are considered in the control algorithm. Controllers can also be classified according to the modeling approach. There can also be model-based controllers that take advantage of analytical models for deriving the controller [24]. Model-free controllers that relay on machine learning methods or use empirical methods can also be used in control of new actuators. There are also hybrid controllers which combine the two approaches mentioned above [24]. Static controllers are time invariant. Their designs are mainly based on beam theory, Cossarat rod theory and the constant curvature model [25,26].

So far, there are no reports in the literature regarding the regulation, and in particular the positioning of drives, in which the silicone/ethanol composite is used. The aim of this work is to present a new design of silicon/ethanol actuator and its investigation results, showcasing its properties. This article also describes the results of research on the positioning of a silicone/ethanol composite-based actuator.

## 2. Materials and Methods

In this paper a prototype actuator based on silicon/ethanol composite is described. It is was initially investigated in an open loop on a dedicated test stand in order to recognize its basic properties and parameters. Controlled cooling with a fan was additionally used to shorten the drive’s return time, after heating. As a following step the P-type and PI-type classical controllers were applied to control the actuator positioning in the closed loop. Here, the step and sinusoidal input signals were used.

### 2.1. Mechanical Design of Actuator

**Figure 1:**
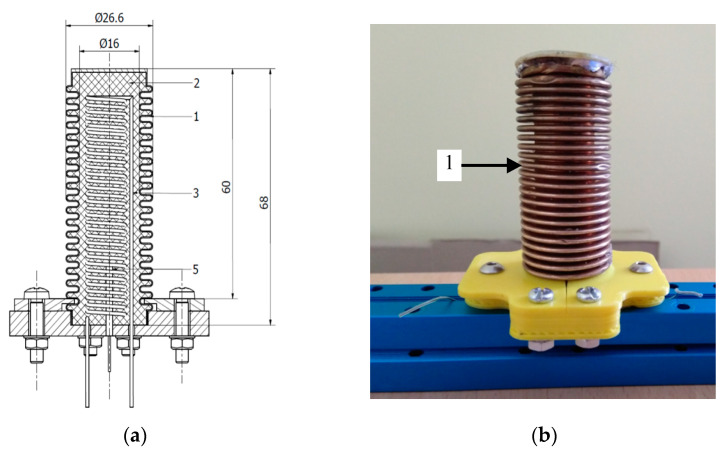
Actuator based on silicon/ethanol composite. (**a**) Design: 1—bellows, 2—composite, 3—heater, 4—thermocouple. (**b**) Prototype.

The actuator, which is a subject of this paper, consists of brass bellows filled with silicon/ethanol composite. The heater (made of kanthal) in the form of coil is placed inside the bellows, together with a thermocouple. The tasks of the bellows are to maintain the shape of the composite and to ensure its return to its initial state during cooling, thanks to its elasticity. The prototype of the actuator is presented in Figure 1. It consists of brass bellows with a bottom internal diameter of 17 mm and an external diameter of 27.5 mm; number of segments in bellows are 19. The dimensions of the actuator bellows are: thickness of the wall equal to 0.3 mm, height of 68 mm and height over base 60 mm. The brass bellows that is commonly applied in pneumatic elements was used. Such bellows are manufactured in various dimensions. The bellows originally did not have a bottom, therefore it was soldered. Before composite solidified, a thermocouple was placed inside the composite, and after solidification the bellows was placed in the recess of the base and blocked with two plates as it is presented in Figure 1.

The heater (made of kanthal), in the form of a coil, is placed inside the bellows, together with a thermocouple. The heater was in the form of a shaft coil hand-wound on a 10 mm screwdriver, centered in the bellows before pouring it with a composite. Heating with the use of Ni-Cr resistance wire has been the most frequently used method in the works described so far. One of the main advantages of this solution is low cost and the simplicity of control. The use of induction heating through a metal housing has been performed [19], however, this method is much more complicated. The resistance of the heater is equal to 13 Ohms. The coil was hand wound up on a 10 mm screwdriver shaft. The bellows with the heater is filled with composite in liquid form that solidifies inside after time. The active material is prepared as composite of 20 vol.% of ethanol and 80 vol.% of silicon, as matrix. The ethanol is commercially available at 99.9% purity and as silicon two part Ecoflex 00-50 is used. The Young module of Ecoflex 00-50 is 0.08 MPa, density is 1.07 g/cm^3^, tensile strength 2.17 MPa [27] and coefficient of thermal expansion (CTE) 275.6 × 10^−4^ K^−1^ [28].

A brass bellows provides additional stiffness to the slender composite core. Thanks to it, the actuator is less prone to buckling under the influence of compression, and therefore it can perform a pushing motion. In addition, the elasticity of the bellows accelerates the return movement of the actuator. In case of earlier actuators consisting of a cylinder filled with a composite and a piston, which also had a pushing motion, there was friction of the composite against the cylinder wall which could disturb the motion. In case of a composite-filled bellows, this problem is now eliminated. Moreover, the shape of the bellows facilitates cooling, which enables the return movement. The elasticity of the bellows limits the elongation of the composite.

Before casting, the composite components are first weighed into the correct amounts and mixed manually for about 5 min. The heater is placed in the center of the bellow and then flooded with the liquid composite. While the composite is still liquid, a thermocouple in a sheath is placed inside it. The composite in the bellows was then left for solidification for 24 h at a room temperature. After solidification of the composite, the bellow is placed in the circular recess of the base and then fixed with two plates as it is shown in Figure 1. Spring constant of the bellow filled with composite and with heater, determined experimentally is equal to 2.5 N/mm. The apparent density of the composite is determined on the basis of geometry and mass measurement of separately cast samples and is 0.9 g/cm^3^. The Young’s modulus of the composite is determined on the basis of the elongation of separately cast samples under load and is 0.03 MPa. In case of a silicone/ethanol composite, there is an effective coefficient of thermal expansion. Its value is not constant and depends on the temperature of the composite. Its value is a function of temperature rise and internal pressure change [29].

### 2.2. Feedback Control of the Actuator

Classical P and PI regulators are used in closed loop control of the built actuator. These regulators are described with the following equations

(1) $$v=K_{p}e\left(k\right)$$

(2) $$v=K_{p}e\left(k\right)+K_{i}\int_{0}^{t}e\left(\tau \right)d\tau$$

where: *v*—value of output, *e*—error, *K_p_*—proportional gain coefficient, *K_i_*—integral gain.

**Figure 2:**
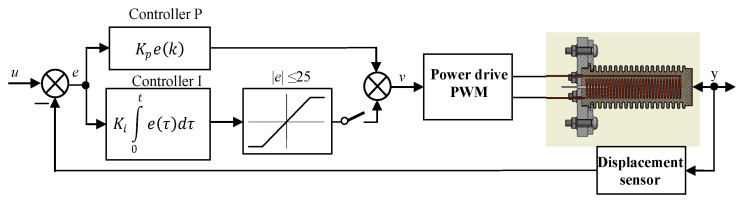
Scheme of the servo drive control system with P and PI controller.

Additionally, in the PI-controller a simple anti-windup block, is implemented, i.e., the controller output signal is saturated to ±25 μm. The scheme block diagram of the controller is presented in Figure 2. The output signals of the regulators are added and given to the power amplifier, which generates the heater supply voltage using pulse width modulation (PWM).

### 2.3. Test Stand

**Figure 3:**
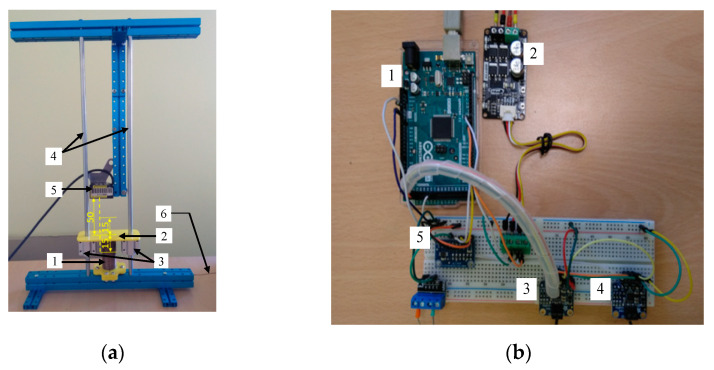
Photos of the test stand: (**a**) station with mounted drive and position sensor; (**b**) electronic control system.

In order to test the actuator, a special test stand has been prepared. It is presented in Figure 3a. The silicon/ethanol actuator (1) is connected to the small platform (2) ends of which are attached to the two linear bearings (3), which may move linearly along the vertical guides (4). This solution provides accurate vertical movement with low friction. The displacement is measured by a laser distance sensor type HG-C1050 (5). The inner temperature of the actuator is measured with a thermocouple type K, which is embedded inside the actuator with only connecting wire visible (6). The mass of all moving parts is equal to 107 g.

**Figure 4:**
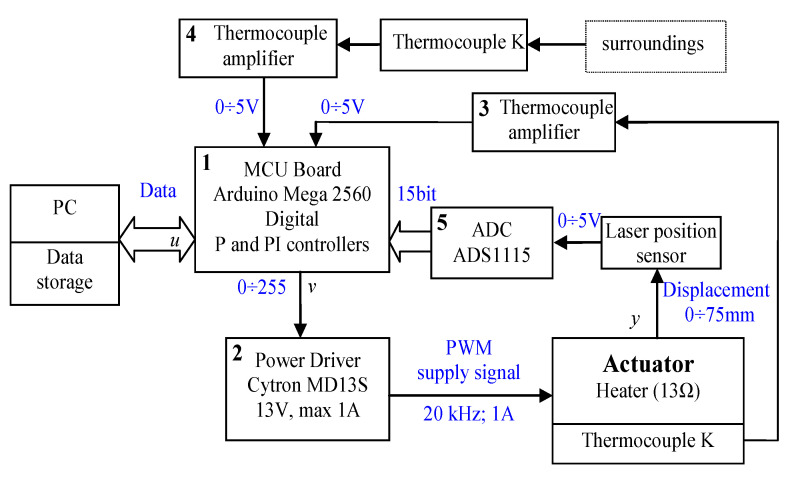
Block scheme of the control and measuring system.

The photo of the control and measuring system is shown in Figure 3b and its block scheme is presented in Figure 4. It is built on the basis of an Arduino Mega 2560 (1). The drive heater is powered by the power drive-Cytron MD13S module (2), which generates the output signal using PWM method. The input signal to the power controller has been established by a control program implemented in the microcontroller based on Arduino platform. Two thermocouples are connected to amplifiers (3) and (4). For actuator displacement measurement the laser distance sensor is used, and its analog output signal is given to the analog to digital converter-ADC (5). In the investigations for distance measurement two laser sensors of Panasonic type HG-C1200 and HG-C1050 were used. The measuring range of the first one was ±80 mm with the measurement center distance equal to 200 mm and its repeatability was equal to 200 µm. The second sensor had measuring range equal to ±15 mm with the measurement center distance equal to 50 mm and with repeatability of 30 µm. Both sensors had analog voltage output with a range of 0÷5 V. They were connected with analog digital converter with reference voltage of 6.144 V that processed the signal with 15-bit resolution. For the sensor type HG-C1200 the resolution of measurement was 6 µm while for the second sensor type HG-C1050 it was 1.13 µm. For measuring the actuator temperature, the thermocouple type K was used. It was connected to the special amplifier for thermocouples. The second thermocouple with amplifier was used to measure ambient temperature.

## 3. Results

In order to determine the basic properties and parameters of the actuator at first the tests in open loop are made. Then the drive controlled in closed loop has been tested.

### 3.1. Step Responses of the Actuator

**Figure 5:**
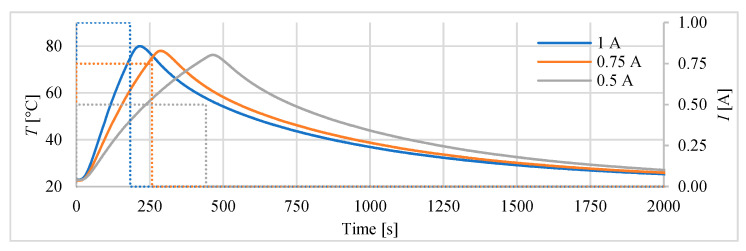
Changes of temperature (supply currents-marked as dashed).

In the first investigations, the heater of the actuator was stepwise switched on, while different values of the supply current were used. During the heating and cooling the temperature inside actuator and the displacements were measured. The investigations have shown that in order to avoid overheating, and as a consequence damaging of the actuator, the temperature of 85 °C should not be exceeded. So, in order to work safety after achieving the temperature of 75 °C, the heating (current) was being switched off and the actuator was cooled by the surrounding environment. Figure 5 presents the recorded changes of temperature inside the actuator in time, for three different supply current.

**Figure 6:**
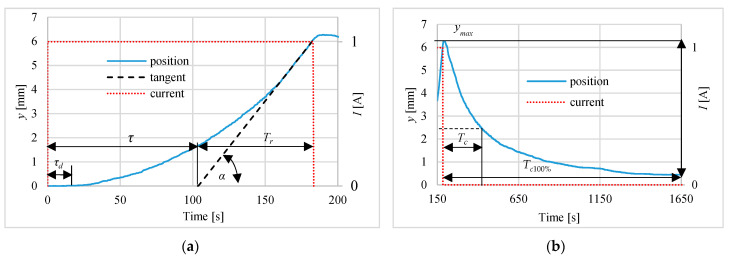
Actuator pulse response for 1 A current during: (**a**) heating, (**b**) cooling.

**Figure 7:**
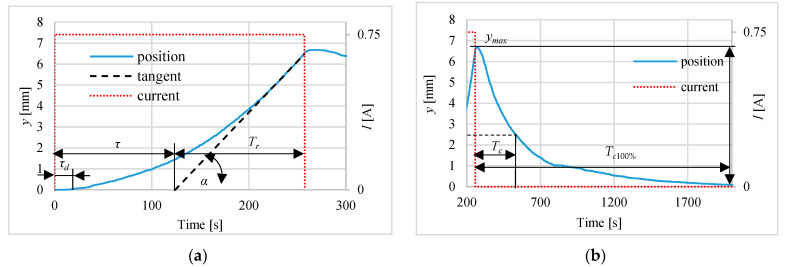
Actuator pulse response for 0.75 A step during: (**a**) heating, (**b**) cooling.

**Figure 8:**
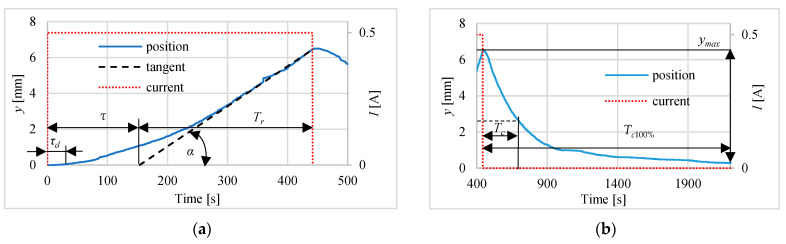
Actuator pulse response for 0.5 A step during: (**a**) heating, (**b**) cooling.

Figure 6, Figure 7 and Figure 8 present the different steps responses of the actuator for the currents equal to: 1.0 A, 0.75 A and 0.5 A. On these figures such parameters as: time of rising *T_r_*, cooling time constant *T_c_* (the time from turning off the current to reaching 0.37*y_max_*) and dead time *τ_d_* are shown. The rising time *T_r_* is determined using the tangent to the step response curve of the actuator, at a point corresponding to a temperature of 75 °C. The parameters *τ* and *T_r_* are used to determine the coefficients of the P and PI regulators. The Table 1 contains the values characterizing the pulse responses parameters. The results show that the dead time is around 13 s, the delay time is between 104 s and 154 s and the rise time varies from 79 s to 288 s in proportion to the amperage and the cooling time constant ranges from 230 to 270 s. The 100% cooling time *T_c_*_100%_ is more than 1500 s.

### 3.2. Closed-Loop Step Responses and Drive Positioning

For all tests of the drive while working in the closed loop control, the maximal current of heating was limited to 1 A. This limitation was achieved by reducing the supply voltage of the power driver to 13 V. During the work, in the closed loop and with the use of P and PI controllers, the input signal *v* given to the power drive was ranging from 0 to 255, which was converted to pulse width and, as a result, a current of 0 to 1 A was given to the actuator. The parameters for these controllers are given in Table 2. They were set experimentally for 0% overshot. The assumed drive position *u* was expressed in micrometers. The current actuator position was measured by laser sensor connected to the 15-bits ADC. The resolution of the measurement was 1.13 μm.

**Figure 9:**
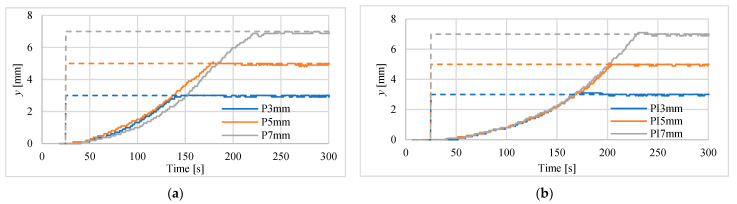
Step responses of the drive, with laser sensor type HG-C1200 controlled by: (**a**) P-type regulator, (**b**) PI-type regulator.

**Figure 10:**
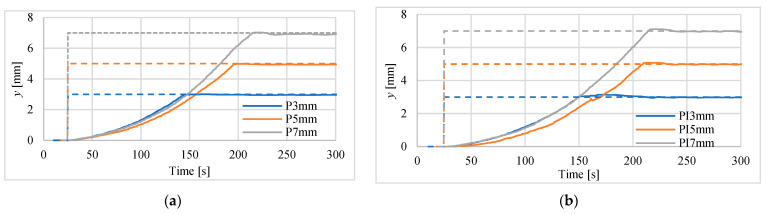
Step responses of the drive with laser sensor type HG-C1050 controlled by: (**a**) P-type regulator, (**b**) PI-type regulator.

The first tests of positioning were made for actuator in vertical position for steps of 3 mm, 5 mm and 7.5 mm. Investigations were carried out using both mentioned above laser sensors. The results for controllers P and PI are presented in Figure 9 and Figure 10. Registered position changes were marked with the first letters denoting the controller type, i.e., P or PI, and step displacements, i.e., 3 mm, 5 mm or 7 mm. In Table 3 the maximum absolute positioning errors for all recorded waveforms and both used laser sensors are listed. The best steady-state (for time > 250 s) positioning results, i.e., error equal to 50 μm, were obtained when the PI controller and the HG-1050 sensor were used.

**Figure 11:**
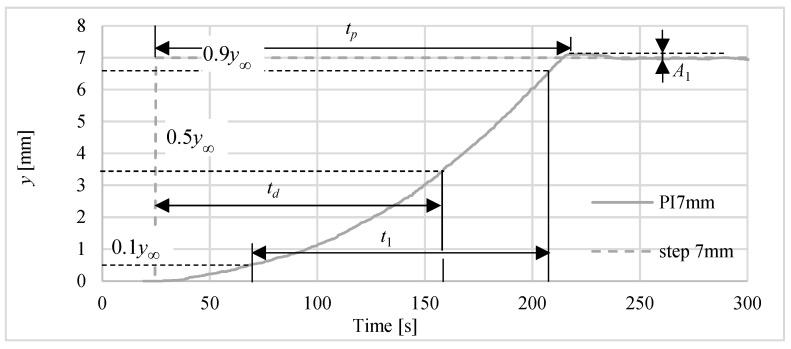
Parameters used for assessment of controllers.

Other parameters used for assessment of positioning process with the use of different controllers are marked in Figure 11. These parameters are: *t_p_*—peak time, *t_d_*—time to reach half of set position, *t*_1_—time to rise from 10% to 90% of the final position, *A*_1_—maximum overshoot in % of final position. An example of determining these parameters for PI controller and for 7 mm set point is shown in Figure 11. The values of these parameters for all investigations are gathered in Table 4. Basing on these collected data, one can notice that the responses for P controller are faster, but PI controller is more accurate. The obtained overshoots are very small, i.e., in most cases below 2%. The results of these studies do not show which of the laser sensors used is better for the dynamics of step responses.

**Figure 12:**
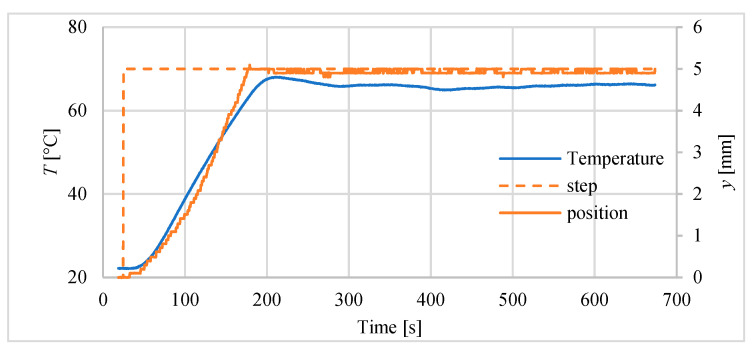
Changes of temperature inside the actuator and its displacement for step input of 5 mm.

The example of temperature changes during positioning is presented for 5 mm step in Figure 12. It can be seen that the displacement curve follows the temperature curve.

**Figure 13:**
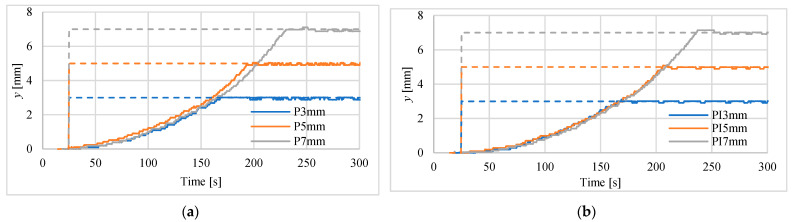
Step responses of the drive with laser sensor type HG-C1200 for actuator in horizontal position controlled by: (**a**) P-type regulator, (**b**) PI-type regulator.

**Figure 14:**
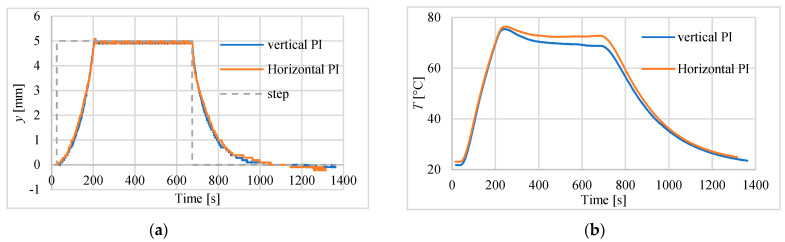
The step responses of the servo drive for actuator positioned vertically and horizontally. (**a**) Position. (**b**) Temperature.

In the next step of investigations the actuator was set in horizontal position, which means that the force of gravity had less influence on the moving parts of the drive. In the tests the laser sensor type HG-C1200 was used. The results are presented in Figure 13 and listed in Table 5. The maximum steady-state errors are very similar to the results obtained when the drive is positioned vertically (see Table 3). For comparison, the Figure 14 shows the behaviors i.e., step responses for vertically and horizontally positioned actuator. The displacement curves do not differ, but in horizontal position, the temperature is slightly higher.

### 3.3. Using a Fan to Reduce Drive Recovery Time

**Figure 15:**
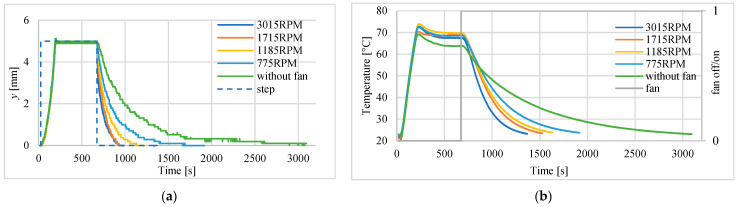
Influence of the speed of fan used during cooling. (**a**) Actuator displacement changes. (**b**) Temperature in the center of actuator.

One of the most important disadvantages of the actuator presented in this paper is long time of cooling which results in long time constant. In order to improve this, the application of forced air flow made by a fan during cooling of the actuator is proposed. To this end, a fan was placed beside the actuator. During the investigations the distance between the actuator and the fan was 5 mm. After the heating was switched off, the fan was turned on and its velocity was set by the controller. The tests were repeated with different rotation speeds of the fan. The influence of the rotation speed on the time constants of returning movement (position changes) are shown in Figure 15a. The decrease of the temperature inside the actuator, for different fan speeds, is presented in Figure 15b. The values of time constant of returning movement are listed in Table 6. It shows that thanks to the use of the fan, the return time constant has decreased from 350 s to 98 s. However, it seems that the fan speed about 1200 RPM can be sufficient to improve the actuator recovery time.

### 3.4. Study of Sinusoidal Signal Follow-Up Control

**Figure 16:**
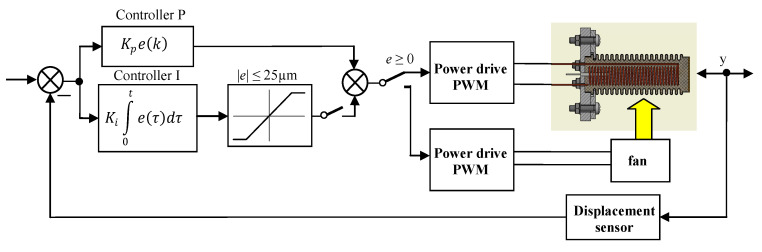
Scheme of P and PI controlled servo drive system with fan.

**Figure 17:**
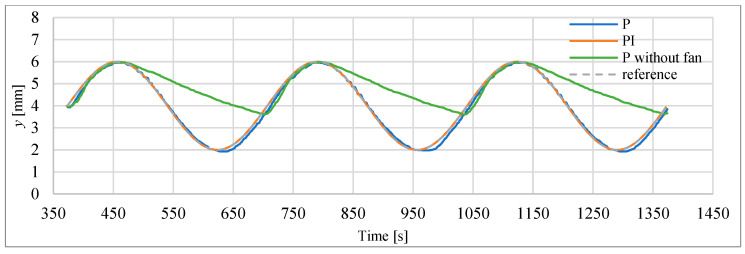
Servo drive tracking of sinusoid signal with frequency of 0.003 Hz.

**Figure 18:**
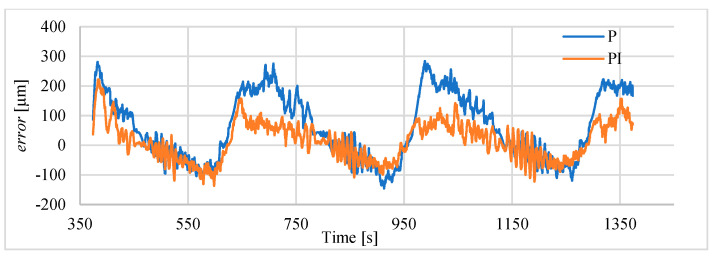
Servo drive tracking error of the sinusoidal signal with the frequency of 0.003 Hz.

The block scheme of the controlled drive after adding the cooling fan to the drive, is presented in Figure 16. In the next investigations, the possibility of tracking sinusoid trajectory of the actuator was investigated. The tests have been carried for stand equipped with laser sensor type HG-C1050. Three input frequencies have been tested. In Figure 17 the results for frequency of 0.003 Hz is presented. Displacement curve of actuator without a fan is also included. It is very clearly visible that, thanks to the use of the fan, a significant reduction in the return time has been achieved, and thus the following of the sine signal has been significantly improved. Figure 18 presents the tracking errors for controllers P and PI. The error for P-type controller vary from about –150 μm to +290 μm and for the PI-type regulator from about –120 μm to +20 μm.

For comparison of the quality of control, the root mean square error (RMSE) and integrated absolute value of error (IAE) were counted according to Equations (3) and (4).

(3) $$RMSE=\sqrt{\frac{1}{n}\overset{n}{\underset{i=1}{\sum }}{\left|y_{ref}\left(i\right)-y\left(i\right)\right|}^{2}}$$

(4) $$IAE=\int_{t=0}^{t=T}\left|y_{ref}\left(i\right)-y\left(i\right)\right|dt$$

Also absolute maximum error was calculated. The parameters used for quality assessment of the control for controllers P and PI and different frequencies are presented at Table 7. They show that the tested servo drive transfers frequencies well up to 0.004 Hz. The maximal absolute error is below 1 mm.

**Figure 19:**
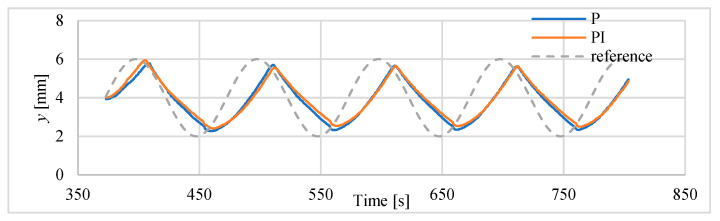
Tracking of sinusoid of 0.01 Hz.

**Figure 20:**
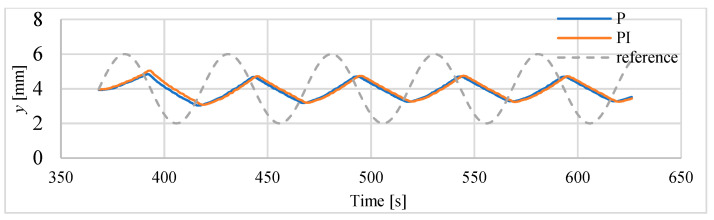
Tracking of sinusoid of 0.02 Hz.

The results of tracking of sinusoids with frequencies of 0.01 Hz and 0.02 Hz are presented in Figure 19 and Figure 20. The parameters of this follow-up control process are summarized in Table 8. In this case, a delay of approx. 12 s is noted, as well as a significant reduction in amplitude and deformation of the sinusoidal shape.

### 3.5. Interference Impact Studies

To ensure that the composite tested was not damaged and worn, it was replaced before the next wave of tests. Verification tests showed that the new drive was not significantly different. In the next series of tests the influence on positioning of disruptive factors has also been investigated. The influence of change of heat dissipation and influence of the change of load have been tested.

**Figure 21:**
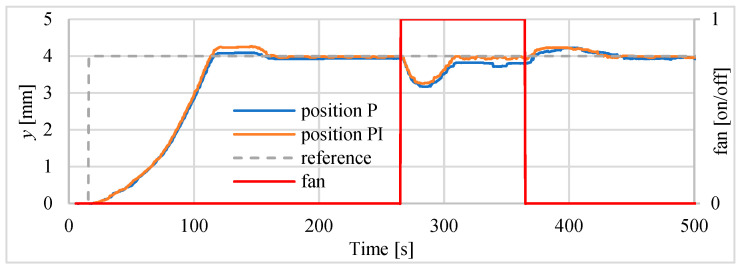
The actuator reaction on switching the fan on and off.

**Figure 22:**
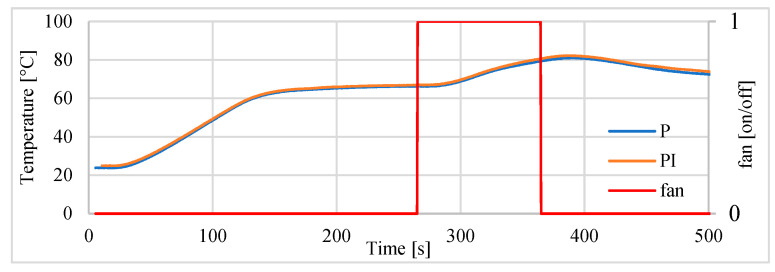
Temperature inside actuator during positioning and during reaction for temporary cooling.

During the first test the drive was controlled by the controller. The position of 4 mm was set to the input of the actuator control unit and, after achieving this distance and after stabilization, a fan was switched on for 100 s. This simulated the rapid change of environment temperature. The velocity of fan was set at 1715 RPM. The displacement was measured using the sensor type HG-C1050. The positioning of actuator driven by P and PI controllers disrupted by action of the fan is presented in Figure 21. It can be seen that the servo drive returns to the set position, however the PI controller is slightly better in this respect. Values of positioning errors are presented in Table 9. The changes of the temperature inside the actuator are presented in Figure 22.

**Figure 23:**
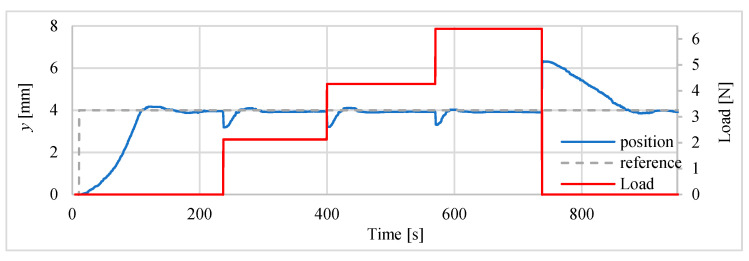
Influence of additional load with controller P.

**Figure 24:**
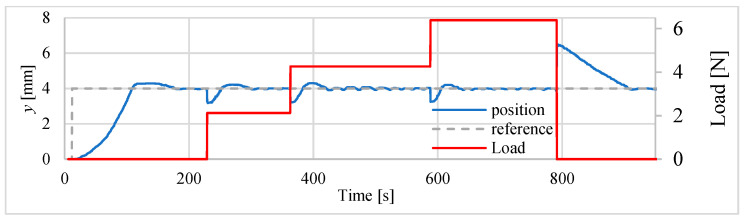
Influence of additional load with controller PI.

During subsequent tests, the vertically positioned drive was been loaded with a mass. In these tests only the heater was controlled by the P and PI controllers. The position of 4 mm was set to servo drive input and then, after about 200 s the drive wasloaded with additional mass. There were 3 loads with masses respectively 216.3 g, 218.1 g and 216.8 g. After adding each of the weights, time was left allowing servo drive to stabilize the position, before adding next element (weight). At the end of the test, the actuator was unloaded. The position was measured with the sensor type HG-C1050. The results of the positioning process (displacement changes) performed after loading the mentioned above masses are presented at Figure 23 and Figure 24. The positioning errors are summarized in the Table 10. The test results show that the load increases the position error from about 30 μm to 100 μm, with the errors being smaller if a PI controller is used.

### 3.6. Investigation of Time Constant during Heating and Cooling with Fan

**Figure 25:**
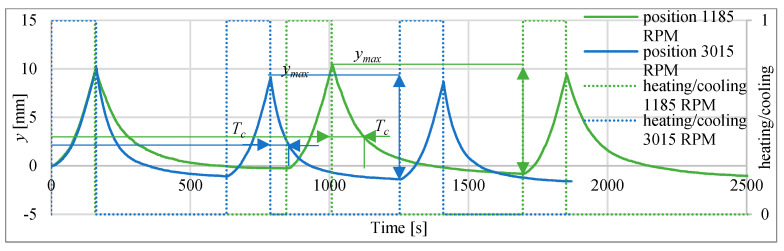
Cyclic heating with 1 A and cooling with fan speed of 1185 RPM and 3015 RPM.

In the investigations the actuator was heated with current 1 A till the temperature achieved 75 °C, then it was cooled using fan until the temperature reached 30 °C and then heated again. Example of the cycle for heating with 1 A and cooling with fan speed of 1185 RPM is presented at Figure 25. The values of time constant are summarized in Table 11. The cooling time constant for fan velocity of 3015 RPM is only 59.6 s.

## 4. Discussion and Conclusions

In this paper an actuator is proposed that is based on silicon/ethanol composite placed in the brass bellows. The main new feature of this solution is a placement of the composite in the brass bellows. As a result, the actuator is less prone to buckling under the influence of compression, and therefore it can perform a pushing motion. In addition, the elasticity of the bellows shortens the recovery time of the actuator. In the solutions described earlier, the actuators consisted of a cylinder filled with a composite and a piston. In these solutions there was friction of the composite against the cylinder wall, which disturbed the motion. The elasticity of the bellows limits the elongation of the composite. In case of composite-filled bellows this problem is eliminated. In this paper also the use of a fan is proposed. Both the shape of the bellows and the fact that it was made of metal that conducts heat well, facilitates the return movement.

Such actuator is later built and investigated in open- and closed-loops. The actuator, test stand and electronic controller are presented. Also, the sensors and signals used in the investigated system are described. In the displacement measurement two different laser sensors i.e., HG-C1200 and HG-C1050 are used. At first the actuator is tested in open loop, i.e., the step responses are recorded. The actuator dead time is around 13 s, the delay time is in a range 104–154 s and the rise time varies from 79 s to 288 s depending on the supply current. So, the dynamics of the proposed actuator are low, but comparable with the dynamics of other temperature activated actuators like TSMA. The cooling time constant, which characterizes the recovery time, is ranging from 230 to 270 s. However, the 100% cooling time *T_c_*_100%_ is more than 1500 s, which is much more than rising time. The research has shown that the actuator step response time parameters are almost the same for both sensors.

In the next step the displacement sensors are used as a feedback signal source. In the closed loop control P and PI controllers are used. Such a system creates a servo drive, which step responses are measured. The investigations results have also shown that the responses for P controller are faster, but PI controller is more accurate. The obtained overshoots are very small, i.e., in most cases below 2%. The values of overshoots are higher for PI controller. The best steady-state (for time > 250 s) positioning results, i.e., error, equal to 50 μm is obtained when the more accurate sensor i.e., HG-C1050 is used. For sensor HG-C1200 the values of less positioning error is 100 μm. The test results show that the load increases the position error from about 30 μm for 0 N load force to 100 μm for 6 N load force, with the errors being smaller if a PI controller is used. The PI controller is better in positioning and is more suitable in presence of disturbances. However, the differences between controllers P and PI are not noticeable.

There are no significant differences in behavior of actuator in vertical and horizontal position without small loads. It can only be noticed that temperatures registered for horizontal position are slightly higher. The reason may be a slight deflection of the actuator causing the heater to be closer to the thermocouple. It is also possible that the temperature inside the actuator is distributed differently than in the vertical position. The load of 6.4 N does not cause significant growth of permanent position error for actuator with controller P and neither for the one with a PI controller. Servo drive is able to compensate disturbances like: ambient temperature and load changes.

The actuator controlled by P or PI regulator is able to follow slow sinusoid signal with frequencies 0.002-0.004 Hz. Significantly better performance in this area is achieved if the fan is applied. For frequencies higher than 0.01 Hz the actuator is too slow, that results in reduction of amplitude and delay.

In the next step of works reported in this paper, the fan is applied for cooling the actuator if the drive position error is negative. The influences of fan rotary velocity on cooling time constant are investigated. Thanks to the use of the fan the recovery time constant is reduced from 350 s to 113 s. The cooling time in cyclic work is reduced to only 59 s. The 100% cooling time *T_c_*_100%_ is reduced from about 1800 s to about 300 s, which means significant improvement of the drive dynamics.

There is an issue of imperfection of the material. The vapors of ethanol slowly escape the material because it is not perfectly hermetic. Moreover areas of material locally overheated stop to work because of destruction of the bubbles. That means that the material adhering to the heater is prone to lose its properties. Due to this fact in firsts cycles, when material is fresh without locally overheated places, the actuator is able to achieve greater elongation than in the later ones.

As the system is not hermetic, ethanol vapor leakage occurs, which can be accelerated in the event of overheating. For this reason, the actuator properties deteriorate over time, and with time the possibility of actuation practically disappears. Ethanol leakage from the silicon/ethanol based actuator remains a vital problem. In the current construction, the leaks through which ethanol is released exist at the connection between the bellows and the base. To prevent escape of vapors of ethanol, the modification of construction of the actuator, especially connection of bellow with a base will be necessary. Moreover sealing of the system should be ensured as a part of the further development of the actuator. It can be achieved by modifying the structure tightening the connection or introducing an additional sealing material.

## Figures and Tables

**Table 1:** Values of parameters describing responses.

*I* [A]	Heating				Cooling
	*τ_d_* [s]	*τ* [s]	*T_r_* [s]	*tag α*	*T_c_* [s]
0.5	13	154	288	0.025	268
0.75	12	124	133	0.049	272
1.00	13	104	79	0.076	232

**Table 2:** Parameters of controllers.

Controller	Parameter	Value
**P**	*K_p_*	0.8
**PI**	*K_p_*	0.93
	*K_i_*	0.003

**Table 3:** Position error for controllers and position.

Sensor	Step	Controller	Max Absolute Error [μm] for *t* > 250 s
**HG-C1200**	3	P	**100**
		PI	100
	5	P	200
		PI	110
	7	P	130
		PI	130
**HG-1050**	3	P	80
		**PI**	**50**
	5	P	70
		**PI**	**50**
	7	P	130
		PI	70

**Table 4:** Step response time and overshot parameters.

Sensor	Step [mm]	Controller	*t_d_* [s]	*t*_1_ [s]	*t_p_* [s]	*A*_1_ [%]
**HG-C1200**	3	P	80	75	**125**	0.40
		PI	104	119	149	3.39
	5	P	102	104	154	3.92
		PI	133	119	179	0.12
	7	P	134	125	230	**0.09**
		PI	154	131	206	1.37
**HG-1050**	3	P	81	85	137	1.44
		PI	86	86	143	5.13
	5	P	121	116	178	0.82
		PI	127	116	187	1.87
	7	P	132	124	191	1.36
		PI	133	146	196	1.96

**Table 5:** Position errors for controllers with sensor type HG-C1200 in horizontal position.

Controller	Step [mm]	Max Error [μm] for *t* > 250 s
**P**	3	120
**PI**	3	110
**P**	5	**100**
**PI**	5	110
**P**	7	120
**PI**	7	140

**Table 6:** Time constant of return movement with cooling by fan.

Fan Velocity [RPM]	Time Constant [s]
**0**	350.4
**775**	163.2
**1185**	119.0
**1715**	112.9
**3015**	98.0

**Table 7:** Quality parameters of step responses.

Frequency [Hz]	Controller	Maximal Absolute Error [mm]	IAE [mm]	RMSE [mm]
**0.002**	P	0.26	112.74	0.10
	PI	0.31	54.92	**0.05**
**0.003**	P	0.29	100.88	0.13
	PI	**0.23**	55.52	0.07
**0.004**	P	0.85	201.76	0.36
	PI	0.90	84.57	**0.17**

**Table 8:** Reduction of amplitude and delay for frequencies 0.01 Hz and 0.02 Hz.

Frequency	Controller	Reduction of Amplitude [mm]		Delay between Higher Amplitudes [s]
		min	max	
0.01 Hz	P	0.222	0.274	12.7
	PI	0.354	0.406	**12.2**
0.02 Hz	P	1.038	1.161	11.9
	PI	0.954	1.083	**12.2**

**Table 9:** Position error before switching on fan, during work of fan and after switching off.

Section	Controller	Error [µm]
**Stabilization after step**	P	70
	PI	**40**
**Stabilization after switching on fan**	P	200
	PI	90
**Stabilization after switching off fan**	P	100
	PI	50

**Table 10:** Influence of load on positioning error.

Section	Controller	Error [µm]
**Without load**	P	60
	PI	**30**
**Load I: 2.1 N**	P	90
	PI	60
**Load II: 4.3 N**	P	90
	PI	60
**Load III: 6.4 N**	P	100
	PI	80
**After removing load**	P	90
	PI	50

**Table 11:** Time constants of actuator return time in different fan velocities.

Fan Velocity [RPM]	Average Time Constant [s]
**0**	272.3
**775**	122.0
**1185**	103.4
**1715**	67.1
**3015**	59.6

## Data Availability

The data that support the findings of this study are available from the corresponding author upon reasonable request.
